# Chemical kinetics and promoted Co-immobilization for efficient catalytic carbonylation of ethylene oxide into methyl 3-hydroxypropionate

**DOI:** 10.3389/fchem.2022.945028

**Published:** 2022-07-22

**Authors:** Jingjie Luo, Pengcheng Liu, Wenhao Yang, Hongyu Niu, Shaojie Li, Changhai Liang

**Affiliations:** Laboratory of Advanced Materials & Catalytic Engineering (AMCE), School of Chemical Engineering, Dalian University of Technology, Panjin, China

**Keywords:** carbonylation, colloid immobilization, ethylene oxide, methyl 3-hydroxypropionate, kinetics

## Abstract

The carbonylative transformation of ethylene oxide (EO) into methyl 3-hydroxypropionate (3-HPM) is a key process for the production of 1,3-propanediol (1,3-PDO), which is currently viewed as one of the most promising monomers and intermediates in polyester and pharmaceuticals industry. In this work, a homogeneous reaction system using commercial Co_2_(CO)_8_ was first studied for the carbonylation of EO to 3-HPM. The catalytic behavior was related to the electronic environment of N on aromatic rings of ligands, where N with rich electron density induced a stronger coordination with Co center and higher EO transformation. A reaction order of 2.1 with respect to EO and 0.3 with respect to CO was unraveled based on the kinetics study. The 3-HPM yield reached 91.2% at only 40°C by Co_2_(CO)_8_ coordinated with 3-hydroxypyridine. However, Co-containing colloid was formed during the reaction, causing the tough separation and impossible recycling of samples. Concerning the sustainable utilization, Co particles immobilized on pre-treated carbon nanotubes (Co/CNT-C) were designed *via* an *in situ* reduced colloid method. It is remarkable that unlike conventional Co/CNT, Co/CNT-C was highly selective toward the transformation of EO to 3-HPM with a specific rate of 52.2 ${\text{mmol·g}}_{\text{Co}}^{-1}{\text{·h}}^{-1}$ , displaying a similar atomic efficiency to that of coordinated Co_2_(CO)_8_. After reaction, the supported Co/CNT-C catalyst could be easily separated from the liquid reaction mixture, leading to a convenient cyclic utilization.

## Introduction

Since the ring-opening carbonylation of epoxide in alcohol solvent has high atomic economy, it is very attractive to prepare downstream high value-added products using epoxide as the reactant *via* catalytic technique (Ocampo and Dolbier, 2004). Among the most commonly used epoxides, ethylene oxide (EO) could be obtained as a petrochemical product in excess capacity by industrial production. It also becomes a promising raw material to produce methyl 3-hydroxypropionate (3-HPM) through carbonylative conversion, with the subsequent hydrogenation product 1,3-propanediol (1,3-PDO) as one of the most expected industrial products for new polyester (PTT) and medical intermediates (Lv et al., 2010; Rajendiran et al., 2018; Wen et al., 2018; Xu and Wu, 2019). No matter how, the industrial path for perspective 3-HPM and 1,3-PDO required the key carbonylative transformation of EO with high efficiency and selectivity, which was, however, greatly challenging until current days.

In the past decades, carbonyl metallic salts have been used as operatable homogeneous catalysts for carbonylation of epoxides. In fact, researchers have reported in detail on the ring expansion and ring-opening carbonylation of epoxides (Suresh et al., 2021). However, complicated homogeneous reaction systems were dominantly used with metastable metal carbonyls with unsatisfied product yields. The early work of Lee and Alper (2004) designed a new Co_2_(CO)_8_/A(6,7-dihydro-5,8-dimethyldibenzo [b,j]-1,10-phenanthroline)/PhCH_2_Br catalyst to synthesize aliphatic polyesters *via* alternating copolymerization of propylene oxide and CO, with 55% yield of atactic polyhydroxybutrate (PHB). While homogeneous reactions were carried out using phosphorus ligands in the carcinogenic benzene under 900 psi, Rowley et al. (2007) reported a special bimetallic [(CITPP)Al(THF)_2_]^+^[Co(CO)_4_]^−^ catalyst efficient in transforming epoxides to β-lactone and subsequently succinic anhydrides *via* one-pot double carbonylation. The rates of both epoxide and lactone carbonylation were suggested independent of CO pressure and are first-order in the catalyst concentration. They also confirmed an opposite solvent effect for the different carbonylation processes.

In fact, Lewis acids as a kind of special ligands coordinating with metal centers were also frequently applied in the epoxide-related reactions. Dunn et al. (2016) reported an improved synthesis of poly (3-hydroxypropionate) (P_3_HP) from ethylene oxide and CO through the intermediate β-propiolactone. The bimetallic [Lewis acid]^+^[Co(CO)_4_]^−^ catalyst was confirmed to be suitable for an optimized carbonylation yield. Maruyama et al. (2022) also reported a selective and quantitative cyclodimerization of an epoxide with a carbonyl compound by Lewis acid–catalyzed processes, where the ring opening of EO was involved as the first step. In a recent work, Zhao et al. (2021) used a series of N,P-ligand–cooperated [LCo(CO)_3_]^+^[Co(CO)_4_]^−^ to catalyze EO (hydromethoxy carbonylation) into 3-HMP and proposed a cooperative reaction mechanism by [Co(CO)_4_]^−^ and its counter cation [LCo(CO)_3_]^+^ (L = Ph_2_PN(*i*Pr)PPh_2_). The yield of 3-HPM largely depended on the type of ligands forming complexes with Co_2_(CO)_8_, and the ion-pair complexes behaved more excellently than the neutral ones.

No matter how, the research progress of Co-based catalysts has stagnated in the past 5 years, and relevant reports still focus on the carbonyl cobalt in a homogeneous reaction system. Although the selectivity for 3-HPM is better, the reaction condition is harsh and in need of thoughtful operation due to CO dissociation and the oxidation of Co^0^. Co_2_(CO)_8_ used as a catalyst is metastable in air and needs to be properly preserved during preparation, storage, and transportation. On the other hand, the separation process is complex, and the homogeneous metal catalyst cannot be fully recovered and reutilized in a simple way, which is not conducive to the sustainable and recycling of resources. Therefore, it is necessary to develop a more stable and durable catalyst system to replace Co_2_(CO)_8_ in advance. In addition, the reaction kinetics of carbonylation of EO to 3-HPM lacks researches in depth and requires further discussion.

Over the years, many studies have been carried out on the carbonylation reaction *via* heterogeneous reactions, such as hydroformylation of olefins and hydroesterification of epoxides by supported noble metals (Li et al., 2003a; Li et al., 2003b; Liu et al., 2009; Liu et al., 2013). Compared with other supports, carbon materials have attracted more attention because of their easily controlled surface specialty and chemical structure. In addition to its outstanding electronic conductivity, carbon materials also exhibit the strong adsorption of CO with manually adjusted surface acidity acting as Lewis acid and Brönsted acid (Kainulainen et al., 1997), which possibly facilitated the immobilization of Co centers as a potential substitution of coordinated Co_2_(CO)_8_.

In this work, Co_2_(CO)_8_ was first used to catalyze the carbonylative transformation of EO to 3-HPM. The reaction parameters including the dosages of reactants; the cooperated ligands and CO pressure were optimized to understand the chemical kinetics of EO carbonylation. In order to promote separation efficiency and sustainable utilization, Co particles supported by pre-treated carbon nanotubes (Co/CNT-C) were synthesized *via* an *in situ* reduced colloid method. It is necessary to mention that the Co/CNT-C sample was selective toward the transformation of EO to 3-HPM with a similar atomic efficiency to that of Co_2_(CO)_8_ cooperated with 3-hydroxypyridine. The key factors for the enhanced catalytic performances were interpreted by combining the initial properties of Co/CNT by using the conventional impregnation method.

## Materials and experimental methodology

### Materials

Co_2_(CO)_8_, pyrazole, 3-hydroxypyridine, and other ligands were purchased from Macklin (A.R.). Ethylene oxide (EO) in tetrahydrofuran (THF) (3.0 mol/L) was purchased from Energy Chemical Co., Ltd. Carbon monoxide with a purity of 99.99% was commercially available and supplied by Shandong Dazhan Nano Materials Co., Ltd. All other reagents were of analytical grade and used as received.

Pre-treated carbon nanotubes (abbreviated as CNT) were used as the supporting material and were preliminarily treated by concentrated HNO_3_ aqueous solution at 120°C for 2 h. The treated CNT was washed with distilled water until the solution was neutral and used as supporting materials after drying overnight.

A solution of 20 wt% Co/CNT-C was synthesized by an *in situ* reduced colloid method, and the process was as follows. A certain amount of sodium lauryl sulfate (SDS), Co(NO_3_)_2_·6H_2_O, and oleic acid with a molar ratio of 10:100:1 were added in water in the presence of 1 g CNT at room temperature. The Co mass loading was kept at 20 wt%. After stirring for 30 min, fresh NaBH_4_ aqueous solution (1 mg/ml) was dropped into the aforementioned mixture with a molar ratio of NaBH_4_ to Co^2+^ as 1:1. The sample powder was obtained after centrifugation, washed, and dried at 90°C. The supported sample went through two-step thermal treatment: calcination at 400°C in air for 3 h and subsequently in H_2_/Ar (v/v = 20/50) at 350°C for 3 h and denoted as Co/CNT-C. An additional Co/CNT-I reference was prepared by incipient wetness impregnation method by impregnating the treated CNT in the aqueous solution of Co(NO_3_)_2_ and aging for 16 h. The following synthesis process (centrifugation, drying, and two-step thermal treatment) was similar to that of Co/CNT-C.

### Characterizations

Transmission electron microscopy (TEM) was performed using a FEI Tecnai G2 F30 electron microscope at an acceleration voltage of 120 KV. Fourier transform infrared (FT-IR) spectroscopy was carried out on a Bruker Vertex 80v FT-IR spectrometer. The specific surface area and pore structure of the samples were investigated using N_2_ physical adsorption/desorption at −196°C using an automatic volumetric sorption analyzer (Quantachrome, Autosorb-iQ-C). X-ray diffraction (XRD) was carried out on a Shimadzu 7000S diffractometer with Cu Kα radiation (*λ* = 1.5418 Å), with a scanning rate of 3^o^/min in range of 10^o^–80^o^. X-ray photoelectron spectroscopy (XPS) was operated on the ESCALAB™ 250Xi (Thermo Fisher Scientific, United States) using the X-ray excitation source of Al Kα. The charge correction is based on C1s peak at 284.6 eV. H_2_ temperature–programmed reduction (H_2_-TPR) was carried out using Quantachrome ChemBET 3000 Chemisorber with the gas flow rate of 30 ml/min. The reduction process was carried out by increasing the temperature from 50°C to 800°C at a heating rate of 10°C/min.

### Carbonylation of ethylene oxide

The carbonylative transformation of EO was carried out in a 50 ml stainless steel autoclave equipped with a magnetic stirrer, temperature control system, and pressure sensing device. In a typical experiment, a certain amount of Co_2_(CO)_8_ and a ligand were mixed with the methanol and EO solution in the autoclave. The aforementioned operation was done in a homemade glove box filled with N_2_ to keep the Co_2_(CO)_8_ stable. The autoclave was then tightened under N_2_ and purged with carbon monoxide three times to evacuate the residual N_2_ in the reactor. After filling the autoclave with CO to a desired pressure, the mixture was heated to a certain temperature (30°C–100°C) under stirring at 800 rpm min^−1^. After maintaining the reaction for a certain period of time, the reactor was cooled down to room temperature and the CO gas was vented off in a fume hood. The reaction mixture was homogeneous and transparent in red-brown. All the catalyst was dissolved in the solution after reaction. It is impossible to separate the catalyst from the reaction mixture by centrifugation. Herein, before further analyzing the reaction mixture by GC, two independent steps were necessary for the separation of catalyst and products. First of all, the reaction mixture was treated continuously by flowing O_2_ at room temperature for 2 h. Purple deposition was observed after the oxidation treatment, and the supernatant liquid became light orange. In the second step, column chromatography was used. An internal standard method was used for quantitative analysis with toluene as the standard. The product mixture was analyzed by gas chromatography using a GC (Agilent 7890B) equipped with a hydrogen flame ion detector (FID) and a capillary column HP-5 (30 m × 0.32 mm × 0.25 μm). Products were also identified using a gas chromatography–mass spectrometer (Thermo Fisher Scientific Trace1300-ISQ LT, United States). The conversion rate of EO and the selectivity of methyl 3-hydroxypropionate (3-HPM) are calculated by the following equations:

$$Conversion\;of\;EO=\frac{n_{0,EO}-n_{t,EO}}{n_{o,EO}}\times 100\text{\%}$$

$$Selectivity\;of\;i=\frac{n_{i}}{n_{0,EO}-n_{t,EO}}\times 100\text{\%}$$

where $n_{0,EO}$ represents the initial mole number of EO; $n_{t,EO}$ represents the molar amount of EO after reaction for a certain time *t*; and $n_{i}$ is the produced mole number of *i* (product).

## Results and discussion

### Carbonylative transformation of ethylene oxide

The carbonylative transformation of ethylene oxide (EO) was catalyzed by using the cobalt catalyst in the presence of CO. Methanol was used in excess as both solvent and reactant. Compared with Co_2_(CO)_8_ as catalyst, inorganic salts such as Co(OAc)_2_ and Co(NO_3_)_2_ displayed poor EO conversion of only 2.3% and 2.9% (Table 1, Entry 1–2), respectively. Also, no methyl 3-hydroxypropionate (3-HPM) was detected in the reaction mixture. The invalid catalytic performances of the inorganic salts should be resulted from the failure coordination between Co and the ligand (Xu and Wu, 2019). If the Co_2_(CO)_8_ was used as the catalyst, the EO conversion easily reached 56.5% at 40°C in 6 MPa CO pressure (Table 1, Entry 3) with 95.8% selectivity toward 3-HPM, in the absence of any ligand. For better catalytic activity, the influences of ligands were studied at first, as listed in Table 1 (Entry 4 and 8–13). A variety of N-donor ligand were used as doping ligands with Co_2_(CO)_8_ for the carbonylative transformation of EO. Among all the tested ligands, 3-hydroxypyridine could be used as a most efficient one with 98.5% selectivity toward 3-HPM at 92.6% EO conversion under only 40°C.

**TABLE 1:** Catalytic performances of Co_2_(CO)_8_ for the carbonylative transformation of EO into 3-HPM at different conditions^a^.

Entry	Ligand	EO conv^b^ (%)	Selectivity (%)		3-HPM yield (%)
			3-HPM	1,2-C_4_H_10_O_2_	
1^c^	n.a.	2.3	0	>99	0
2^c^	n.a.	2.9	0	>99	0
3	n.a.	56.5	95.7	0	54.1
4	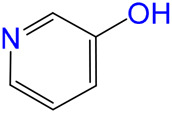	92.6	98.5	1.5	91.2
5^d^		92.8	78.9	21.3	73.2
6^d^		93.6	87.5	12.5	81.9
7^d^		94.1	85.8	14.3	80.7
8	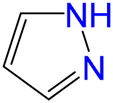	84.8	97.1	2.9	82.3
9	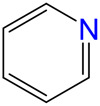	73.7	96.7	3.3	71.3
10	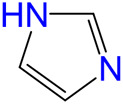	73.0	96.8	3.2	70.7
11	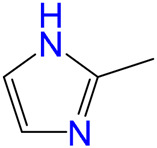	54.7	95.6	4.4	52.3
12	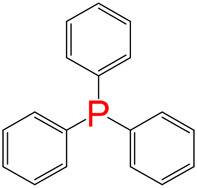	24.2	90.5	9.5	21.9
13	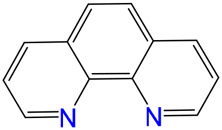	26.1	92.0	8.0	24.0

a Reaction condition: Co_2_(CO)_8_. at 40°C in 6 MPa CO for 4 h; Co/ligand = 1:2; 3.0 mol/L EO in THF, balanced with 16 ml methanol, if not specified.

b Conversion of EO.

c Reaction catalyzed by Co(OAc)_2_·4H_2_O for Entry 1 and Co(NO_3_)_2_·6H_2_O for Entry 2.

d Reaction pressure of Entry 5–7 was 4, 6, and 8 MPa at 80°C for 8 h.

In fact, Co_2_(CO)_8_ coordinated with the N-donor ligands to form Co-centered complexes in different paths depending on the specific electronic environment. Cobalt has an empty d-orbital and is easily complexed with the ligand by lone pair electrons on N. The nucleophilicity of a particular N atom in these ligands determines their interaction with Co as well as the subsequent catalytic performances. Compared to pyridine, the -OH group on the pyridine ring of 3-hydroxypyridine resulted in rich electron density on the aromatic ring due to p–π conjugated effect. The lone pair electrons on N may accounts for a stronger interaction between N and Co atoms in the complexes. The N-groups on pyrazole and imidazole with the $\pi_{5}^{6}$ structure are similar to that on the pyridine ring, and the position of an additional −NH group further influences the electronic environment on the pyrrole ring. It can be revealed that the catalytic performances of Co_2_(CO)_8_ largely depended on the electronic environment of N atoms on the aromatic rings. In fact, proton transfer between cobalt carbonyl and the N-containing compounds was commonly reported by researchers (Kristjansdottir et al., 1991). Zeng et al. (2021) also assumed that an acid–base neutralization easily happened between the HCo(CO)_4_ with 3-hydroxypyridine. It was thus inferred that a positive interaction may exist between the N-donor with rich electron density and the Co center, correlated to the better EO transformation. The much lower EO conversion *via* Co_2_(CO)_8_ in the presence of 1,10-phenanthroline may be caused by the steric hindrance to coordinate with the Co center atoms. Triphenylphosphine was also used as a typical P-donor for comparison, which was less active and selective for 3-HPM compared to most of the N-donor ligands. Liu et al. (2007) suggested that appropriate N-containing compound easily interrupted the Co-Co bond in Co_2_(CO)_8_ to form the active $\text{Co}{\left(\text{CO}\right)}_{4}^{-}$ anion. Other research work also confirmed that acidic compound generated from the alcoholysis of carbonyl cobalt salt easily resulted in undesired ethers. Herein, N-donor ligand, especially the 3-hydroxypyridine, acted as base additives stabilized the catalyst and partially neutralized the catalyst acidity, inhibiting the etherification by-products (Liu et al., 2006; Gorbunov et al., 2019; Zeng et al., 2021).

**FIGURE 1:**
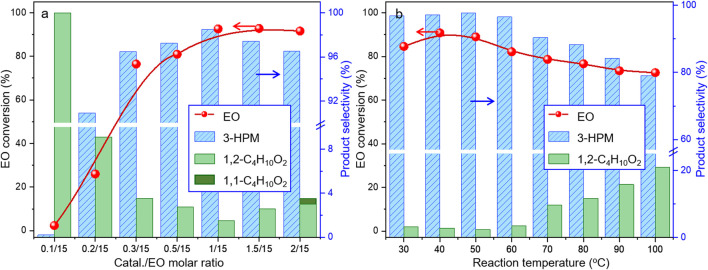
Catalytic performances of Co_2_(CO)_8_ for carbonylative transformation of EO as a function of **(A)** molar ratio between Co and EO at 40°C and **(B)** reaction temperature. Reaction condition: 6 MPa CO for 4 h; Co/3-hydroxypyridine = 1:2; and Co/EO = 1/15, balanced with methanol, 1,1-C_4_H_10_O_2_:1,1-Dimethoxyethane, and 1,2-C_4_H_10_O_2_:1,2-Dimethoxyethane.

Under such circumstance, transformation of EO was further optimized by Co_2_(CO)_8_ in the presence of 3-hydroxypyridine. The experimental data suggested that the increase of CO pressure under the same temperature correlated with very slightly increases of EO conversion and 3-HPM selectivity (Table 1, Entry 5–7). The EO conversion and product selectivity change as a function of the Co/EO molar ratio was also studied, as shown in Figure 1A. The EO conversion was only 2.4% with the Co/EO ratio of 0.1/15, and most of the product was 1,2-dimethoxyethane (selectivity > 99%) as a result of nucleophilic addition without any participation of CO molecules (Kim et al., 2006). Both the EO conversion and 3-HMP selectivity grew logarithmically along with the Co/EO ratio. The EO conversion reached 92.6% with 98.5% 3-HPM selectivity at Co/EO of 1/15. Further increasing the ratio of cobalt catalyst, the selectivity of 3-HPM decreased while 1,2-dimethoxyethane (2.8%) was produced with trace amount of 1,1-dimethoxyethane (1.0% selectivity), although the EO conversion was maintained. It was suggested that CO molecules could be inserted into the skeleton of carbon chains to form 3-HPM only if the catalyst dosage kept in an appropriate Co/EO range.

The influence of reaction temperature was also investigated, as illustrated in Figure 1B. The EO conversion displayed a parabolic trend with the temperature, and 84.6% EO could be successfully transformed at only 30°C under 6 MPa CO with 96.8% selectivity toward 3-HPM. The catalytic performance maximized at 40°C, while the subsequent increasing of temperature induced the steadily decease of both the EO conversion and 3-HPM selectivity. The selectivity of by-product 1,2-dimethoxyethane increased to 20.9% at 100°C. At lower temperature (<60°C), the nucleophilic reaction was facilitated between EO and MtOH with the increasing temperature (Zeng et al., 2021). However, it is a common sense that the catalytic performance increases until the kinetic equilibrium, only if a stable catalyst, was in use (except for a third-order reaction). Herein, it is inferred that the as-formed complexes by Co_2_(CO)_8_ and N-donor ligand may encounter thermal decomposition under higher reaction temperature.

### Kinetics of ethylene oxide carbonylation

**FIGURE 2:**
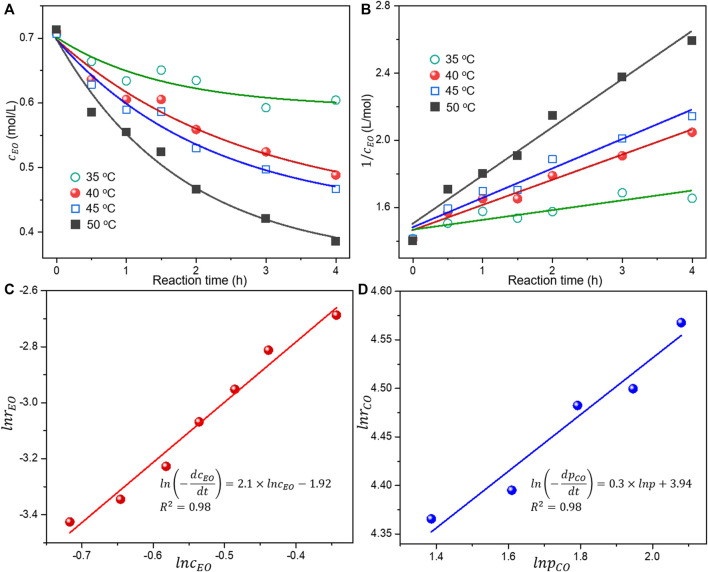
**(A)** Reaction data of the EO concentration at different reaction time and temperature, **(B)** linear relationship between $1/c_{EO}$ and reaction time according to the second-order reaction kinetics, **(C)** linear relationship between $lnr_{EO}$ and $lnc_{EO}$ at 40°C of the EO carbonylation, and **(D)** linear relationship between $lnr_{EO}$ and $lnp_{CO}$ for the EO carbonylation using Co_2_(CO)_8_. Reaction condition: 35°C–50°C, 6.0 MPa CO, 800 rpm for 4 h, Co/3-hydroxypyridine = 1:2, and Co/EO = 0.2/15, balanced with 16 ml methanol, if not specified.

To the best of our knowledge, few researches were reported concerning the chemical kinetics for EO carbonylation, which is essential to suffciently understand the catalytic performances. In order to understand the key reaction factors and the kinetics of EO carbonylation, the concentration of EO changing as a function of reaction time at different temperature was studied, as shown in Figure 2A.

The conversion of EO was kept at 10%–30% by adjusting the conditions to make a precise estimation of the reaction order and other kinetic parameters. The EO was steadily consumed during the reaction and its concentration monotonously decreased with the reaction time. A higher reaction temperature in the range of 35°C–50°C resulted in the faster transformation of EO as indicated by the lower EO concentration detected under the same reaction time. The reaction data was subsequently fitted by the zero-order, first-order, and second-order reaction models in Figure 2B and Supplementary Figure S1 (supporting information). The corresponding R^2^ and rate constant (k_A_) for each linear relationship are listed in Supplementary Table S1. A much higher R^2^ approaching 1 based on the experimental data suggested the reaction order concerning EO was approximately 2. The reaction constant rapidly increased with the temperature, confirming the much accelerated reaction rate under higher temperature in the range of 35°C–50°C. For better interpretation, the reaction rate equation can be written in terms of EO concentration and CO pressure as Eq. 1. The influences of methanol on the reaction rate could be ignored in Eq. 1, since methanol is used as both reactant and solvent in excessive quantity.

$$r_{EO}=-\frac{dc_{EO}}{dt}=k_{A}c_{EO}^{\alpha }P_{CO}^{\beta }$$ (1)

The parameter $k_{A}$ is the rate constant with respect to EO, while α and β represent the reaction orders with respect to EO and CO. To obtain the reaction order of EO (*α*), CO pressure was kept sufficient and consistent at 6.0 MPa. Herein, the term concerning the CO pressure ( $P_{CO}^{\beta }$ ) can be viewed as a constant value during the reaction. The simplified Eq. 2 can be expressed as follows:

$$r_{EO}=k_{A}c_{EO}^{\alpha }P_{CO}^{\beta }=Kc_{EO}^{\alpha }$$ (2)

$$lnr_{EO}=lnK+\alpha lnc_{EO}$$ (3)

The reaction order (*α*) in terms of EO was thus obtained by the slope of a linear relationship between the logarithm of EO concentration and the logarithm of the reaction rate (Figure 2C). The mathematics fitting result suggested an apparent reaction order of 2.1 with respect to EO, further confirming the previous fitting results.

$$r_{CO}=k_{CO}c_{EO}^{\alpha }P_{\text{CO}}^{\beta }=K^{\prime }\;P_{CO}^{\beta }$$ (4)

$$lnr_{CO}=lnK^{\prime }+\beta lnp_{CO}$$ (5)

The reaction order (β) in terms of CO can be detected based on Eqs 4,5 under low EO conversion (superfluous EO concentration with nearly consistent quantity during reaction). As revealed by the slope of $lnr_{CO}$ vs. $lnp_{CO}$ in Figure 2D, the reaction order with respect to CO was 0.3. The low reaction order of CO suggested the weak dependency of the reaction rate with CO pressure. The pressure of CO in process was found to have a limited effect on the catalytic activity for carbonylation compared with the EO concentration. Kramer et al. (2006) reported the excellent production of β-lactones produced *via* carbonylation at only atmospheric CO pressure. Even for the CO inserting into the cobalt-alkyl bond process, Church et al. (2006) reported an independent reaction rate of CO pressure over the range considered. These phenomena may account for the very slight variation of EO conversion as the CO pressure changes (Table 1, Entry 4–7).

According to the aforementioned investigation on kinetics, the differential rate equation could be written in the following form:

$$\begin{array}{c} r_{A}=-\frac{dc_{A}}{dt}=k_{A}c_{EO}^{2}c_{CO}^{0.05} \end{array}$$ (6)

The activation energy (E_a_) of the catalytic system reflected the necessary energy barrier to activate the reactant molecules and can be viewed as a parameter describing the catalytic efficiency (Joshi et al., 2018; Nazimov et al., 2021). According to the Arrhenius equation, the Ea based on Co_2_(CO)_8_ using 3-hydroxypyridine as a ligand can be estimated. The linear relationship between *lnk*_A_ and the reciprocal of *T* in K is shown in Supplementary Figure S2. The Ea value calculated by the slope of the Arrhenius curve is 62.9 kJ/mol. It is necessary to mention that such value was occasionally similar to the reported energy barrier for disproportionation of Co_2_(CO)_8_ to [Co(MeOH)_6_][Co(Co)_4_]_2_ in methanol under carbonylation reactions (Mirbach and Mirbach, 1985). It is inferred that the formation of Co-centered complex may become an indispensable course for an efficient EO transformation.

**FIGURE 3:**
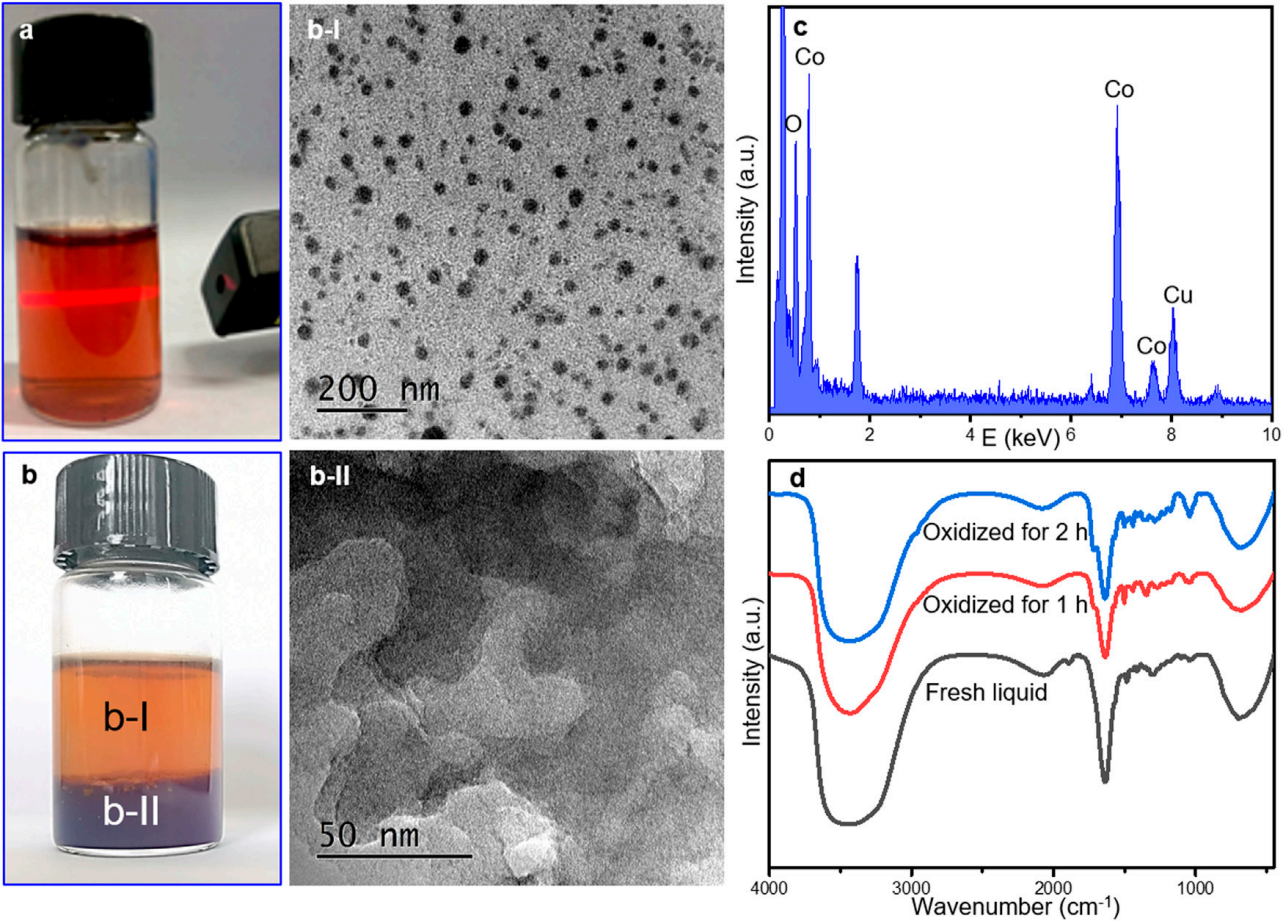
Electronic pictures of fresh mixture extracted immediately after reaction **(A)** and treated by oxidization with pure O_2_ at room temperature for 2 h **(B)**, **(B-I)**, and **(B-II)** illustrated the TEM images of supernatant liquid and under-layer deposition in b. **(C)** TEM/EDX analysis of the B-II sample, and **(D)** FT-IR spectra of fresh mixture after reaction without and with O_2_ flowing at room temperature.

For better confirmation, the solid catalysts before and after reaction were characterized by FT-IR spectra. It has to be noted that separation of the catalyst from the reaction mixture was indeed a tough task before any other analysis of the products. The cobalt complex was uniformly dissolved in the liquid and a seemingly homogeneous solution was obtained after reaction, where metal colloid was generated as confirmed by the Tyndall phenomenon (showing a characteristic light path) in Figure 3A. Colloidal Co-containing particles may exist in the reaction mixture.

It is impossible to separate the catalyst directly by centrifugation or column chromatography. Herein, an additional re-oxidation step was operated to recycle the cobalt-containing composition. The fresh liquid after reaction was subsequently treated by the flowing O_2_ for 2 h continuously to oxidize the Co colloid into Co-containing deposition (Figure 3B). The TEM images of the transparent solution in Figure 3A displayed the well dispersed particles as shown in Figure 3B-I, indicating the as-formed Co colloidal particles protected by organic compounds. The under-layer deposition was also collected and tested by TEM and EDX analysis. Amorphous CoO_x_ was detected as a main composition (Figures 3B-II,C).

FT-IR spectra of the fresh mixture after reaction without and with oxidation treatment were displayed in Figure 3D. Infrared spectroscopy is one of most useful methods for characterizing the presence of different bands. The dominant peaks of -OH stretching and -CH deformation vibration at approximately 3,400 cm^−1^ and 692 cm^−1^ were observed in all samples (Li et al., 2012; AlOmar et al., 2016). The adsorption peaks at 1,644 cm^−1^ and 1,724 cm^−1^ originated from the aromatic skeleton vibration and the stretching vibration of the C=O bond in the coordinated cobalt catalyst (Xie and Wan, 2018), while the peak at 1,888 cm^−1^ suggests the presence of $\text{Co}{\left(\text{CO}\right)}_{4}^{-}$ species, which disappeared rapidly after 1-h oxidation. After oxidation, *ν*_C-O_ = 1,050 cm^−1^ attributed to the C-O stretching vibration (Qiu et al., 2021) became apparent while peak situated at 1,888 cm^−1^ disappeared, confirming the possible decomposition of $\text{Co}{\left(\text{CO}\right)}_{4}^{-}$ species and the variation of functional groups after oxidation and separation procedures.

No matter how, the high efficiency of Co_2_(CO)_8_ collaborated with 3-hydroxypyridine happened at the expense of pseudo-homogeneous reaction with untoward separation of the reaction liquid. It makes the separation of the metal catalyst and the recycle more difficult to be operated with considerable cost, which is also a big concern for the industrial production unit. Research works focusing on the carbonylative transformation of different chemical compounds by supported catalysts were also studied in recent years. However, tough reaction conditions including high temperature and pressure were requisite for better EO conversion and 3-HPM selectivity. The leaching of metal species was also one of the challenges during the liquid-phase heterogeneous reactions. Considering the *in situ* formed Co colloid within the reaction process as well as the potential advantages of the easily controlled carbon surface, a specific sample with cobalt active centers supported by CNT were herein synthesized *via* an *in situ* reduced ***C*** olloid method and denoted as Co/CNT-C. A reference synthesized by conventional ***I*** mpregnation was also synthesized for comparison and denoted as Co/CNT-I.

**FIGURE 4:**
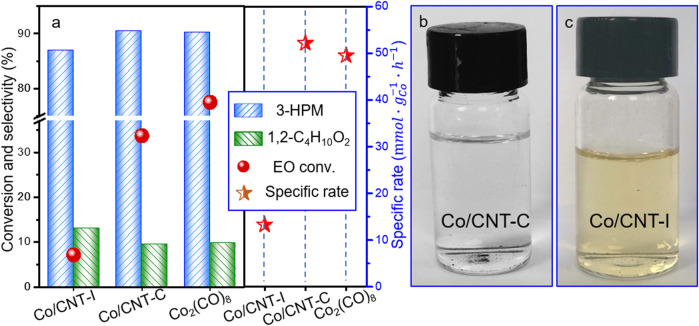
**(A)** Catalytic performances and specific rates and **(B,C)** electronic of the up-layer solution by centrifugation after reaction using Co/CNT-C and Co/CNT-I. Reaction condition: 60°C, 5 MPa CO for 5 h; Co/EO = 0.3/15 (0.1 g catalyst) for 20 wt% Co/CNT-I or Co/CNT-C; and Co/3-hydroxypyridine = 1:2, Co/EO = 1/15 for Co_2_(CO)_8_, balanced with methanol.

It is remarkable that compared to the poor EO conversion by Co/CNT-I (7.2%), EO conversion of 33.7% could be reached using Co/CNT-C by the *in situ* reduced colloid method. The EO conversion was 77.5% in the presence of Co_2_(CO)_8_ with 3-hydroxypyridine as co-catalyst at the same condition. The selectivity toward 3-HPM was 92.5% by Co/CNT-C and slightly higher than that using Co_2_(CO)_8_. Concerning the excessive Co content used in the Co_2_(CO)_8_ system, the specific rate based on per gram Co per hour was calculated, as displayed in Figure 4A. The Co/CNT-I synthesized by conventional impregnation was only 13.3 ${\text{mmol·g}}_{\text{Co}}^{-1}{\text{·h}}^{-1}$ , while Co_2_(CO)_8_ and Co/CNT-C had close specific rates. The specific rate by supported Co/CNT-C was 52.2 $\text{mmol}{\text{.g}}_{\text{Co}}^{-1}{\text{.h}}^{-1}$ and even higher than that by Co_2_(CO)_8_ (49.6 $\text{m}mol.g_{Co}^{-1}.h^{-1}$ ). The better capacity to transform EO molecules by supported Co/CNT-C in terms of the same amount of Co centers could be disentangled. It has to be mentioned that the Co/CNT-C after reaction could be conveniently separated by centrifugation as illustrated in Figure 4B. The up-layer liquid was transparent with very light yellow, and colorless solution could be obtained and injected into GC for precise analysis after simple filtration. Meanwhile, the reaction mixture using Co/CNT-I by conventional impregnation method displayed a common metal leaching phenomenon as revealed by the yellow solution in Figure 4C. The advantageous stability of Co/CNT-C during reaction could be unraveled preliminarily.

**FIGURE 5:**
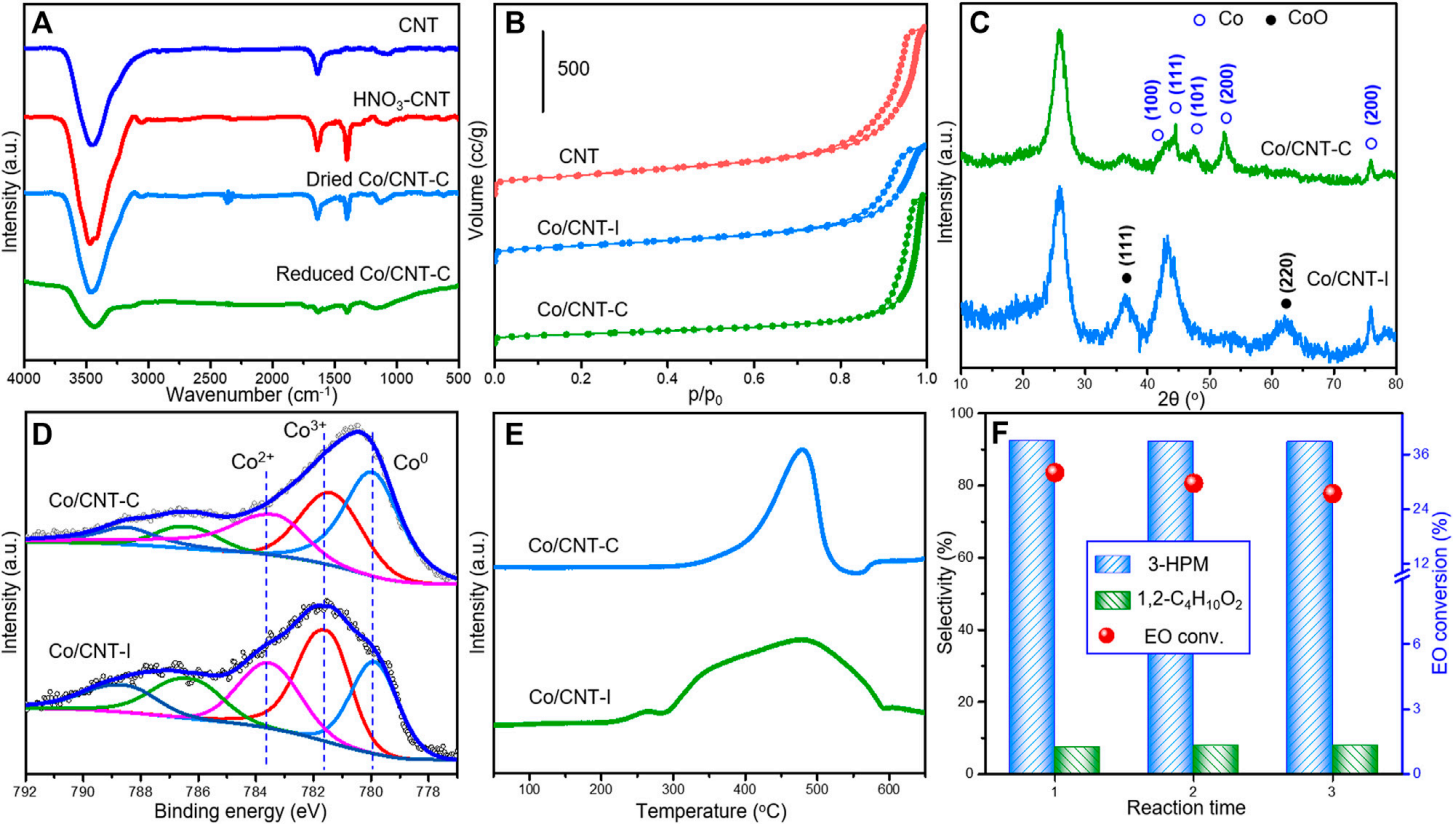
**(A)** FT-IR spectra of Co/CNT-C during the synthesis process, **(B)** N_2_ adsorption–desorption curves, **(C)** XRD patterns, **(D)** XPS spectra of Co 3*d* core level, **(E)** H_2_-TPR profiles of Co/CNT-C and Co/CNT-I by different methods, and **(F)** Cycle tests by Co/CNT-C. Reaction condition: 60°C, 5 MPa CO for 5 h; Co/EO = 0.3/15 (0.1 g catalyst), balanced with methanol.

For better information, the FT-IR spectra of different Co/CNT-C and precursors during the synthesis are studied in Figure 5A to understanding the formation of surface functional groups on these samples. Nitric acid–treated CNT was used as the supporting material. The adsorption peak at 1,640 cm^−1^ was ascribed to the aromatic skeleton vibration in the main structure of CNT. After HNO_3_ treatment, large amount of carboxyl species was generated as revealed by the C-O stretching vibration and O-H deformation vibration at with adsorption peak at 1,400 cm^−1^. The deposition of Co particles caused the obvious decreased ratio of the later adsorption peak, suggesting the decreasing number of oxygenated groups during metal loading. Our previous research confirmed the possible consumption of surface oxygenated groups during tight metal loading (Luo et al., 2017; Luo et al., 2020). After the two-step thermal treatment, the adsorption peaks in the reduced Co/CNT-C were further exhausted.

The N_2_ adsorption–desorption curves of CNT and supported Co/CNT (Figure 5B) displayed the typical type IV isotherms, suggesting the maintained mesoporous structure after Co loading. Compared with the initial surface area of CNT (310.14 m^2^/g), the surface areas of Co/CNT-I and Co/CNT-C decreased to 221.8 and 204.6 m^2^/g, respectively. It could be attributed to the blocking of pores during the loading and growth of Co particles. Concerning the greatly differed catalytic performances of Co/CNT-I and Co/CNT-C, the XRD patterns in Figure 5C gave a clear evidence of the distinctive structures by different synthesis. Large amount of Co and CoO coexisted in Co/CNT-I, while both the diffraction peaks of Co and CoO became broadened in the Co/CNT-C, suggesting the smaller crystal sizes of Co-containing particles in such sample. The TEM image of Co/CNT-C is displayed in Supplementary Figure S3 to understand the surface dispersion of Co. Co particles existed as small black dots (Supplementary Figure S3A) on the surface of CNT. There was not any clear aggregation of Co particles. The HAADF-STEM EDX-elemental mapping of the Co/CNT-C also suggested the high dispersion of Co species on the detected area. It is also remarkable to note that Co^0^ was more readily enriched on the surface of Co/CNT-C and accounted for 44.3% of the total surface Co species by the Co 3 ***d*** XPS core level (Figure 5D). In the Co/CNT-I reference, only 20.6% Co^0^ dispersed on the surface. The much lower H_2_ consumption by Co/CNT-C (2,500 μmol/g) than Co/CNT-I (3815 μmol/g) during H_2_-TPR (Figure 5E) further confirmed the accumulation of oxidative Co species in the Co/CNT-I. The combination results of XRD and XPS suggested a higher dispersion of Co particles with more Co^0^ sites exposed on the surface, which may correlated with the faster reaction rate *via* Co/CNT-C samples as reported by many carbonylation research works using supported metal catalysts (Xu et al., 2011; Zhang, 2012; Ibrahim and Denmark, 2018; Wang et al., 2021). The Co/CNT-C was further tested as a recycle catalyst for the EO carbonylation. After three reaction cycles, the conversion of EO in Figure 5F were still kept at 27.4% with the stabilized high selectivity of 3-HPM (92.1%). The discussion about Co/CNT-C suggested that it is a stable catalyst with nice specific rate based on the same Co atomic efficiency of Co_2_(CO)_8_ while avoiding the costly and complicated separation/post-treatments based on the metastable Co_2_(CO)_8_ system.

## Conclusion

In this work, carbonylative transformation of EO was investigated using commercial Co_2_(CO)_8_ in a homogeneous system to produce 3-HPM. The reaction parameters including dosages of reactants, cooperated ligands, and CO pressure were optimized to understand the chemical kinetics of EO carbonylation. The reaction rate was interpreted as second-order in EO concentration and less sensitive to the CO pressure. However, Co-containing colloid was generated during the reaction, causing the tough separation process before any further chemical analysis. Concerning the impressive separation and sustainable utilization problems caused by the homogeneous reaction process, colloidal Co particles were directly supported on pre-oxidized CNT (Co/CNT-C) *via* an *in situ* reduced colloid method. Greatly improved EO conversion was observed *via* Co/CNT-C, if compared with conventional Co catalyst. The better activity was ascribed to the high dispersion of Co particles with good Co^0^ surface exposure and suitable amount of Co^3+^ as Lewis acid sites. The specific rate based on per gram Co per hour *via* Co/CNT-C was 52.2 $\text{mmol}{\text{.g}}_{\text{Co}}^{-1}{\text{.h}}^{-1}$ , although the macroscopical EO conversion still needs work in progress. The Co/CNT-C sample was highly selective toward 3-HPM with selectivity of 92.5% and a similar atomic efficiency to that of Co_2_(CO)_8_ cooperated with 3-hydroxypyridine. Most important of all, the immobilized Co particles on Co/CNT-C enabled the facile separation and recycling for potential applications of EO carbonylation.

## Data Availability

The raw data supporting the conclusion of this article will be made available by the authors, without undue reservation.
