# Supplementary material for: Chemical kinetics and promoted Co-immobilization for efficient catalytic carbonylation of ethylene oxide into methyl 3-hydroxypropionate
Source: Front Chem. 2022 Jul 22;10:945028. doi: 10.3389/fchem.2022.945028 (PMC9354985; doi:10.3389/fchem.2022.945028)
Supplement: Supplementary file 1 [file DataSheet1.PDF]

Supplementary Material

1 Supplementary Data

Figure S1. Linear relationship between (a) c_{EO} and reaction time according to zero-order reaction kinetics, and (b) lnc_{EO} and reaction time according to first-order reaction kinetics over a temperature range of 35-50 ^{o}C, 6.0 MPa CO, 800 rpm for 4 h, Co/3-hydroxypyridine = 1: 2, Co/EO = 0.2/15, balanced with 16 mL methanol.

Figure S2. Arrhenius plot for the carbonylative transformation of EO by $Co_2(CO)_8$ using 3-hydroxypyridine as ligand in the temperature range of 30-50 ^{o}C.

FigureS3. Typical TEM image and HAADF-STEM EDX-elemental mapping of Co/CNT-C.

Table S1. The R^2 and k_A for mathematics fitting of kinetic reaction models during the EO carbonylation.[a]

2 Supplementary Figures and Tables

2.1 Supplementary Figures

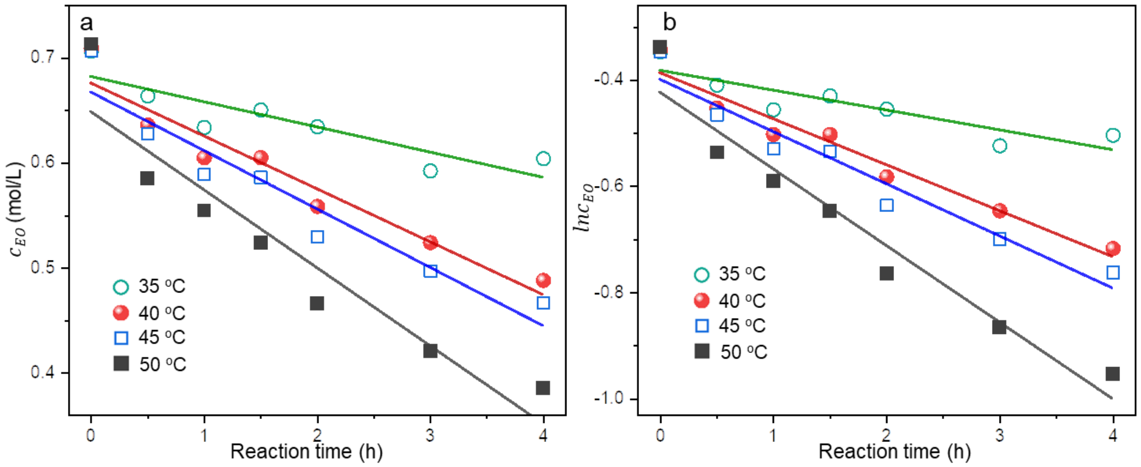

Supplementary Figure 1. Linear relationship between (a) c_{EO} and reaction time according to zero-order reaction kinetics, and (b) lnc_{EO} and reaction time according to first-order reaction kinetics over a temperature range of 35-50 ^{o}C, 6.0 MPa CO, 800 rpm for 4 h, Co/3-hydroxypyridine = 1: 2, Co/EO = 0.2/15, balanced with 16 mL methanol.

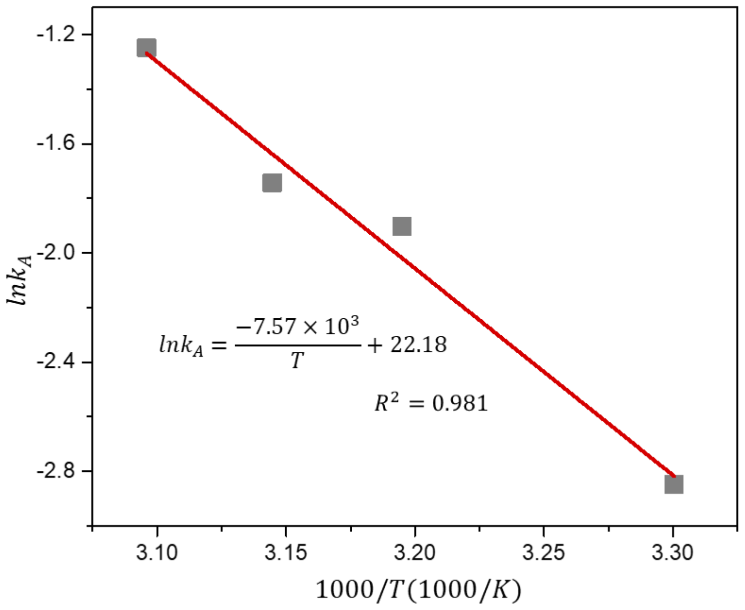

Supplementary Figure 2. Arrhenius plot for the carbonylative transformation of EO by $Co_2(CO)_8$ using 3-hydroxypyridine as ligand in the temperature range of 30-50 °C.

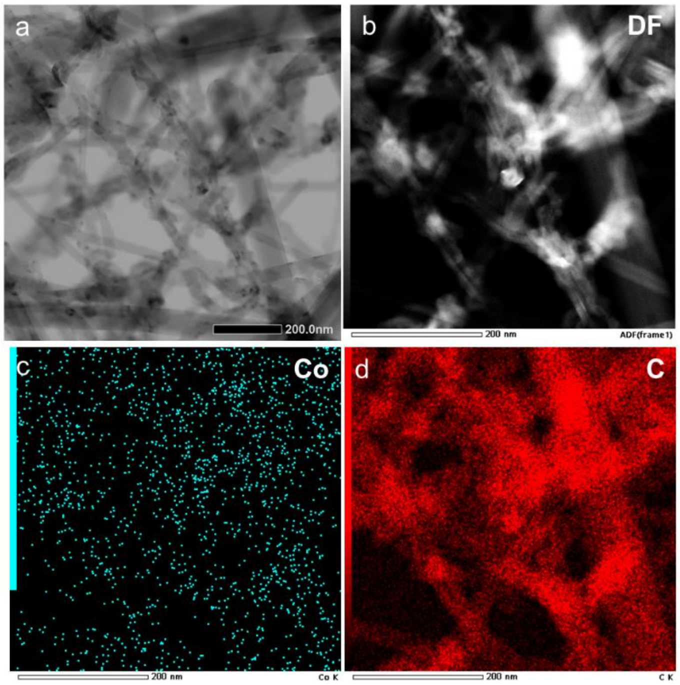

Supplementary Figure 3 Typical TEM image and HAADF-STEM EDX-elemental mapping of Co/CNT-C.

Supplementary Table 1. The R^2 and k_A for mathematics fitting of kinetic reaction models during the EO carbonylation.[a]

Reaction order	35 °C		40 °C		45 °C		50 °C	
	R^2	k_A	R^2	k_A	R^2	k_A	R^2	k_A
Zero-order	0.79	0.024	0.93	0.050	0.92	0.056	0.89	0.074
First-order	0.79	0.382	0.96	0.387	0.94	0.399	0.94	0.423
Second-order	0.81	0.058	0.97	0.149	0.97	0.175	0.98	0.287

[a] Experiments carried out over a temperature range of 35-50 °C, 6.0 MPa H_2, 800 rpm for 4 h, Co/3-hydroxypyridine = 1: 2, Co/EO = 0.2/15, balanced with 16 mL methanol.
